# The needle and the haystack: single molecule tracking to probe the transcription factor search in eukaryotes

**DOI:** 10.1042/BST20200709

**Published:** 2021-05-18

**Authors:** Matteo Mazzocca, Tom Fillot, Alessia Loffreda, Daniela Gnani, Davide Mazza

**Affiliations:** Experimental Imaging Center, IRCCS San Raffaele Scientific Institute, Milan 20132, Italy

**Keywords:** search mechanism, single molecule, transcription factors

## Abstract

Transcription factors (TFs) regulate transcription of their target genes by identifying and binding to regulatory regions of the genome among billions of potential non-specific decoy sites, a task that is often presented as a ‘needle in the haystack’ challenge. The TF search process is now well understood in bacteria, but its characterization in eukaryotes needs to account for the complex organization of the nuclear environment. Here we review how live-cell single molecule tracking is starting to shed light on the TF search mechanism in the eukaryotic cell and we outline the future challenges to tackle in order to understand how nuclear organization modulates the TF search process in physiological and pathological conditions.

## Introduction

Transcription Factors (TFs) control the expression of their target genes by recognizing specific sequences in regulatory elements (REs) of chromosomal DNA at the single base-pair level. If we idealize the explored environment as a well-stirred non-crowded solution, the time *τ_search_* taken by a TF to reach a target of size *a* within a volume *V* via unhindered random three-dimensional (3D) diffusion (with diffusion coefficient *D*) is given by the Smoluchowski relationship for diffusion-limited reactions [1,2]:

$$\tau_{search}=\frac{V}{4\pi Da}$$

**Figure 1.**
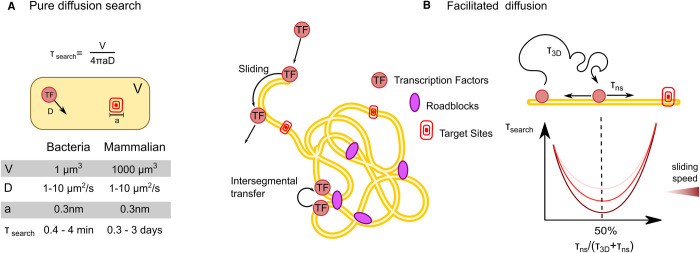
Classical search strategies. (**A**) Diffusion limited search. Shown is the Smoluchowski relationship for diffusion limited reactions, where *τ_search_* is the time taken by a TF to find a target site, V is the size of the explored volume, a is the target site (typically assumed as the size of a nucleotide, since the TF needs to align to its target with base-pair precision and D is the diffusion coefficient of the molecule). Typical expected values for these paramters are provided in the table. (**B**) The mechanism of facilitated diffusion speed-up the search process, with an efficiency that depends on the fraction of time the TF spend sliding on DNA.

In this simplified scenario, *τ_search_* values would range between minutes in bacteria to days for mammalian TFs (Figure 1A), numbers that appear incompatible with the rapid transcriptional responses observed in living cells [3]. Starting from the 70s, pioneering *in vitro* studies evidenced that bacterial TFs can find their targets orders of magnitude faster than the Smoluchowski limit, and this discovery stimulated the investigation of an alternative model to describe the TF search, named facilitated diffusion [4,5].

Facilitated diffusion is an intermittent search process (Figure 1B), where the TF alternates between non-exhaustive 3D diffusion and local sampling of DNA [2,6]. 3D diffusion allows the TF to cover large distances in short time. Local sampling, achieved through one-dimensional (1D) diffusion on DNA (sliding) and/or intersegmental transfer mediated by non-specific TF–DNA interactions, results in an exhaustive search of the sampled ‘DNA domain’. Such intermittent approach can speed up the search process because instead of searching for a sequence with base-pair precision, the TF would look for a larger target (i.e. the stretch of DNA that will be then sampled exhaustively via non-specific interactions) [7]. The maximization of the search efficiency requires a fine balance of the time spent bound non-specifically to DNA, since a limited sliding phase would neutralize the benefits of local scanning, while an excess of it could segregate the TF away of its target sites. Theoretically, the optimal balance is achieved when the TF spends 50% of its time bound non-specifically to DNA [6]. Following the proposal of the facilitated diffusion model, multiple DNA binding proteins have been shown to be capable of 1D sliding and intersegmental transfer on DNA *in vitro* [2,4,8–11]. Notably, by single-molecule imaging of fluorescently labeled LacI in living *E. coli* [12], and by inserting artificial ‘roadblocks’ in proximity of the TF binding sites, Elf and colleagues provided the first direct evidence that facilitated diffusion speeds the TF target search mechanism *in vivo*, but to a lesser extent than the optimal theoretical condition. Indeed, LacI spends >90% of its time bound non-specifically, and visits the same binding site several times before binding to it, highlighting a potential trade-off between search speed and search accuracy [13]. Strikingly, it has been recently demonstrated that many other bacterial DNA binding proteins spend the majority of their time bound to non-specific sites on DNA *in vivo* [14].

The progress in characterizing how eukaryotic TFs navigate the nucleus is still in its early phases. Here, the long search times predicted by the Smoluchowski limit might be alleviated by having many TF copies searching for their targets simultaneously, but whether the TF copy number is sufficient to explain the rapid recruitment dynamics observed at promoters (in some instances as short as tens of seconds [15]) is unclear. Furthermore, whether the search processes contributes to determining the subsets of genes that the TF can find and regulate (the TF ‘target selectivity’ problem [16]) is one of the many questions that await answers: Do eukaryotic TFs perform facilitated diffusion? Do different TFs display different search mechanisms? Do TFs adapt their search strategy during development and disease? What is the role of the nuclear organization in shaping TF dynamics? Here we discuss how live-cell single molecule tracking (SMT) can be used to contribute answering these still open questions.

## Intranuclear single molecule tracking: analyzing TF binding

**Figure 2.**
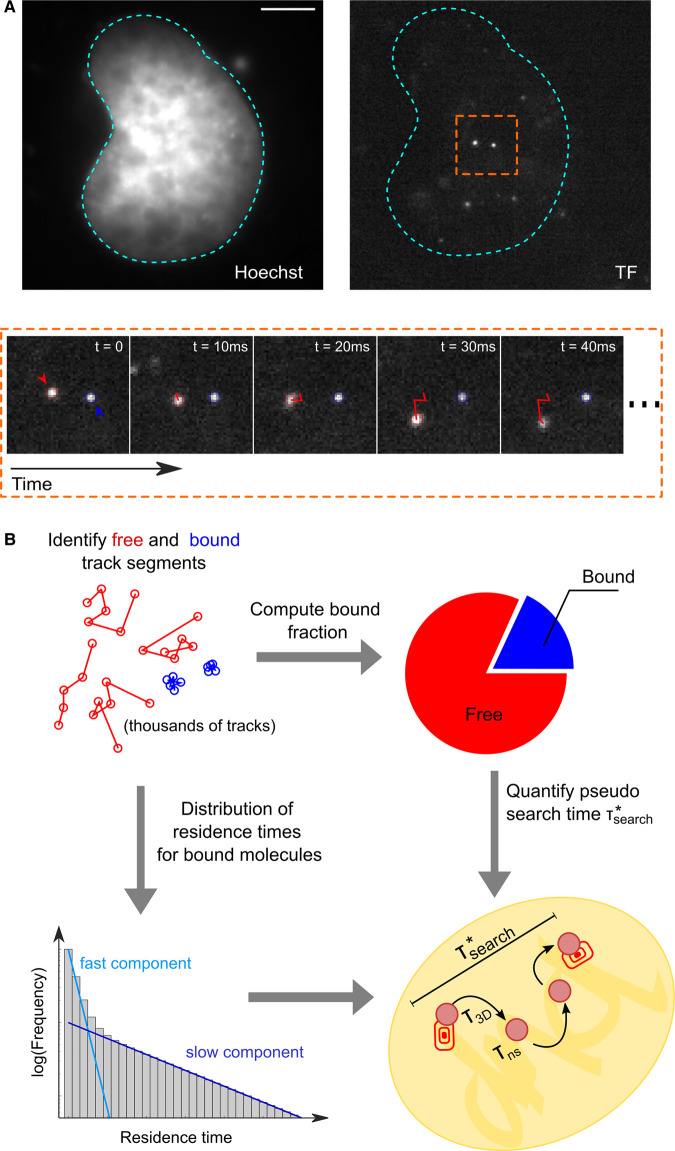
Intranuclear SMT to quantify search times. (**A**) in SMT the diffraction limited spots corresponding to individual TF molecules in the nucleus are tracked over time. (**B**) The single-molecule tracks, that typically last for few frames are segmented into ‘bound’ and ‘free’ molecules and analyzed in terms of the distribution of residence times and fraction of segments belonging to each state. These parameters are then combined to estimate the ‘pseudo-search time $\tau {*}_{search}$, that is the time a TF molecule spends on average between two consecutive binding events (at any site).

SMT is a fluorescence microscopy technique requiring the sparse labeling of a protein of interest with bright and photostable fluorescent dyes, so that individual molecules appear as diffraction-limited spots, that are then fit by a 2D-Gaussian distribution, to determine their position with sub-pixel precision [17,18]. By ‘connecting the dots’ in consecutive frames [19,20] (Figure 2A), it is possible to quantify the dynamics of the tagged protein at the individual molecule level with nanometric precision, to identify different populations of molecules displaying different dynamic behaviors and to estimate the kinetic rates of switching between these states. While historically associated to the characterization of membrane protein dynamics, SMT has emerged in the last decade as one of the methods of choice to quantify protein motion in the nucleus [21–23], thanks to the development of novel fluorescent labeling approaches [24,25] and to the improvement of widefield-based illumination schemes allowing for optical sectioning [26,27].

While it would be desirable to directly measure the search time by tracking the TF molecule from the moment in which it enters the nucleus to when it reaches a selected locus, photobleaching limits our capability of tracking single molecules for prolonged time. From a practical point of view, photobleaching defines the average number of distinct images that we can collect from a single molecule [22]. By tuning the ‘blind times’ between exposures it is possible to trade temporal resolution for track duration: measurements of diffusion properties and/or population quota (e.g. the TF bound fraction) are typically performed at a high frame rate to minimize mistracking [28]. Oppositely, measurements of residence time are often performed at slower frame rates, to limit the impact of photobleaching [29,30].

Chromatin appears as slowly mobile [31], with diffusion coefficients <0.1 µm^2^/s. Therefore, the measurement of the quota of TF molecules bound on chromatin (the TF bound fraction) is carried out by isolating the track segments corresponding to quasi-immobile molecules (Figure 2B), using methods ranging from kinetic modeling of the distribution of displacements [22,28] to track classification using Hidden Markow models [32,33] and machine learning approaches [34]. Analysis of mutated TF versions is then typically used to confirm that the identified molecules are indeed bound to DNA [29,35,36].

Following isolation of the bound segments, the next step is to quantify the duration of TF/DNA interactions. Confounding effects brought by slowly diffusing molecules [22,29] and by photobleaching [37] have led to multiple methods to extract the distribution of residence times from SMT data, ranging from long-exposure acquisitions to blur out non-chromatin bound molecules [29], to multi-temporal scale acquisitions, to correct for bleaching [21,38]. Despite these methodological differences, the resulting distribution of residence times is often analyzed in terms of a multi-exponential decay (Figure 2B). While most analysis of this type preventively assumes just two types of binding events, interpreted as non-specific and specific binding, respectively [29,30,39,40], recent work is providing a more complex scenario. Hypothesis-free modeling is showing that the residence time distribution of some TFs can be described by many more components than two (up to six, [41]). Furthermore, at least some exogenous [42] and endogenous [43] TFs can display a non-exponential power-law distribution of residence times, blurring out the distinction between specific and non-specific events. At the moment, it is unclear whether the observed differences are biological (different factors behaving differently) or technical (caused by the different methods used to acquire, correct and analyze the residence times distribution, [37]). Therefore caution is recommended when comparing residence time measurements obtained by different groups on different proteins.

Importantly, if non-specifically and specifically bound populations are well-separated, the aforementioned parameters (the average residence times, their relative abundance and the estimated bound fraction) can be combined to calculate a ‘pseudo’-search time $\tau_{search}^{*}$, that a TF, after leaving a specific target, spends to find a second one, or in other words the time between two specific binding events [29,39,44,45] (Figure 2B). $\tau_{search}^{*}$ estimates are particularly useful when comparing the search efficiency of a TF in different conditions (e.g. before and after its activation) or to compare the search process of different TFs all analyzed using the same experimental settings.

## Intranuclear single-molecule tracking: analyzing TF diffusion

The analysis of diffusion is also important to characterize the TF search: macromolecular crowding [46] and transient non-specific interactions [47] of the TF with DNA or other nuclear structures can result in the slowdown of the TF. For example, this is the case for LacI diffusion in bacteria [12], whose non-specific interactions with DNA last only 5 ms — faster than the typical acquisition rate of SMT movies — but are so frequent that result in an apparent 90% reduction in the TF diffusion coefficient. Remarkably, the duration and the extent of transient binding might be TF- and context- specific in eukaryotes, as highlighted by screening the non-specific binding of hundreds of mammalian TFs to mitotic chromosomes [48].

**Figure 3.**
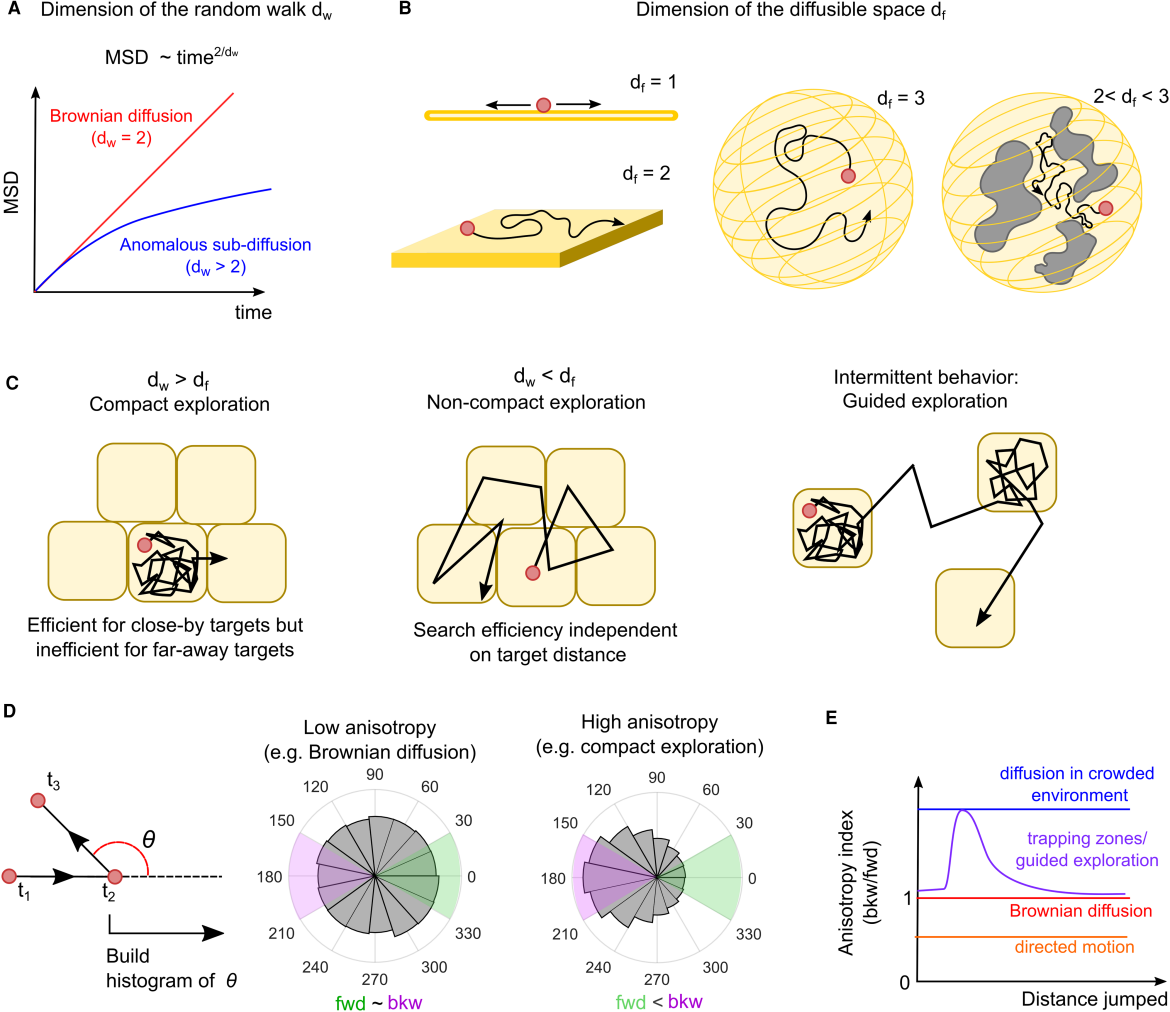
Exploration strategies in the eukaryotic cell nucleus. The TF exploration strategy can be determined by two numbers, the walk dimension *d_w_* and the diffusible space dimension *d_f_*. (**A**) The MSD analysis of the diffusing molecules can be used to quantify *d_w_*, that is equal to 2 for Brownian diffusion and larger than 2 for hindered (anomalous) diffusion. (**B**) *d_f_* describes the space that can be explored by the TF, and it is equal to 1, for molecules sliding on DNA, 2 for molecules exploring surfaces, 3 for molecules diffusing freely in 3D. If the diffusible space is obstructed on multiple scales, *d_f_* can be smaller than 3. (**C**) When *d_w_* > *d_f_*, the exploration is compact and the TF visits all the sites of a region of space before leaving it. Oppositely, when *d_w_* > *d_f_* the exploration is non-compact and the TF samples the potential targets only sparsely. Compact and non-compact exploration can be combined together intermittently, and this combination can guide the TF molecules to their target sites speeding up the search process. (**D**) The anisotropy of the diffusion process (measured as the distribution of angles between consecutive displacements) can inform on the search strategy, as compact exploration should result in high backward anisotropy. (**E**) Measuring the diffusional anisotropy as function of the distance jumped by the molecules can reveal exploration modes, including the presence of trapping zones that underlie guided exploration.

The influence exerted by the nucleus on the search mechanism can be unveiled also by the trajectory that the TF follows in the diffusion process. For instance, macromolecular complexes can hinder to the TF motion, forcing the TF to change direction. In case of sliding instead, the TF trajectory is dictated by the DNA fiber that provides sort of a road for the TF motion. The sum of all these constraints to diffusion ultimately determine how the TF explores the nuclear space and -importantly- the efficiency of the search mechanism. In this respect, the exploration strategies can all be reduced into two universal classes, i.e. *compact* vs *non-compact exploration* [49,50] (Figure 3A–C).

Compact exploration occurs when the molecule has a high probability to completely explore a region of space before leaving it. Once again, an example is provided by 1D sliding. Instead, non-compact exploration occurs when the searching molecule typically leaves a certain region before having explored it completely. In this case, the TF diffuses in a scattered manner throughout the space, leaving many sites unvisited [49]. A typical example of non-compact exploration is provided by free diffusion in an unobstructed 3D environment, as in the Smoluchowski limit.

While compact exploration is an efficient search strategy if the target is located close to the initial position of the searching molecule, the efficiency of non-compact exploration is independent on the distance, and therefore can provide a mean to rapidly escape from regions poor of targets. According to this scheme, the facilitated diffusion process can be idealized as an intermittent process where the TF rapidly finds its targets by alternating between a compact (1D sliding) and a non-compact exploration (3D diffusion) phase. Other mechanisms can however give rise to compact exploration, ranging from molecular crowding to trapping of the TF in specific nuclear zones/compartments. Given the heterogeneity of the nuclear environment (see below), it is possible that the TFs could alternate between these two phases. Such intermittent behavior — recently dubbed *guided exploration* [51] — generalizes the facilitated diffusion model and could speed up the search efficiency (Figure 3C).

The measurement of the ‘compactness' of a search process only relies on two numbers: the dimension of the random walk *d_w_* (Figure 3A) and the dimension of the space that can be explored by the molecule *d_f_* (Figure 3B)(e.g. *d_f_* = 1 for 1D sliding, *d_f_* = 3 for unhindered diffusion in a volume, 2 < *d_f_* < 3 in comparments filled with ‘obstacles’ to diffusion, ‘fractal’ environments, etc.). When *d_f_* > *d_w_* the exploration is non-compact, while when *d_f_* < *d_w_* the exploration is compact. Although *d_w_* can be readily extracted by the mean squared displacement (MSD) analysis of SMT data, the measurement of *d_f_* is more complex and requires either high 3D resolution images of chromatin organization [52], or detailed rheology measurements [53].

Nevertheless, the exploration process can be indirectly investigated by analyzing the statistical properties of the diffusing TFs trajectories. Repeated revisiting of the same spatial region implies the TF walking back on its steps: by applying this concept, compact exploration can be inferred by analyzing the anisotropy of the diffusion process — accumulated over many TF tracks — or in other words how frequently a diffusing TF molecule walks back on its steps. The analysis of anisotropy (Figure 3D) can also inform on the underlying mechanism of such compact exploration: for example, diffusion in a crowded ‘fractal’ environment would cause TF diffusion to be anisotropic at every spatial and temporal scale [54], while local trapping would display as anisotropic diffusion only at the spatial scale corresponding to the size of the traps [51] (Figure 3E).

## Do eukaryotic TFs perform facilitated diffusion in living cells? Is the search mechanism shared among different TFs? Is the search mechanism modulated upon TF activation?

Some mammalian TFs have been shown to perform 1D diffusion on naked DNA *in vitro*, but the direct observation of sliding within a living eukaryotic nucleus is challenging, because it is expected to occur over few hundreds of base-pairs, corresponding to tens of nanometers at most, comparable to the resolution limit of single-molecule microscopy approaches [18]. Evidence for a role of facilitated diffusion in mammalian cells has therefore been obtained only by combining *in vitro* measurements of 1D diffusion on DNA and *in vivo* SMT estimates of kinetic parameters. One example is the tumor suppressor p53, whose *in vitro* sliding is mediated by its unstructured C-terminal domain [55]. Accordingly, deletion of the p53 CTD results in a reduced fraction of long-lived binding events in living cells [22]. Similarly, Sox2 has been shown to slide *in vitro*, and this could explain how the same TF performs its search in relative short times and with high targeting efficiency [29]. Clearly, the connection of these fast search processes with the 1D diffusion observed *in vitro* are purely speculative, and possibly other mechanisms like the guided exploration mediated by local trapping described above could be at play to speed the target search up.

Also, how much these observations can be generalized to other TFs is unclear. While most of eukaryotic TFs belongs to just a few classes with rather stereotyped domains involved in specific DNA binding [3], the protein regions responsible for non-specific interactions with DNA and compact exploration are often unstructured and more variable across different TFs [56,57]. Thus, it is likely that different TFs could be ‘guided’ differently by the nuclear environment, and consequently display different search strategies. For instance, MSD and diffusional anisotropy analysis has been used to determine that the TF c-Myc largely explores the nucleus in a non-compact manner, while the nuclear kinase P-TEFb undergoes compact exploration [54]. More recently, an impressive SMT analysis in yeasts of the 10 members of the preinitiaton complex (PIC), showed that the different general transcription factors (GTFs) composing the PIC display signatures of compact exploration to different degrees. Additionally, the inhibition of key factors of the PIC, such as Mediator and PolII modulates the subdiffusive behavior of other GTFs [44]. Some members of the PIC might therefore guide others to the target sites, and such hierarchical guiding might pose the bases of the step-wise assembly of the PIC at the promoters.

Related to the last point, it is also worth to ask whether a single TF can modulate its search mechanism in response to some physiological or pathological cues, to modulate its *target selectivity* and/or to globally control the occupancy of its target genes. Indeed, association rates of TFs to chromatin can be modulated by post-translational modifications [58,59] or by activation by ligands [30]. Recent results on MITF suggest that low-affinity non-specific binding sites can out-compete specific regulatory elements, and that the equilibrium between specific and non-specific binding can be tuned by post-translational modifications of the TF residues that make contacts with the DNA phosphate backbone [60]. The physical properties of the nucleus can also be modulated to control the search process. For example, the decrease in nuclear volume observed during the early development phases of the zebrafish embryo [61] results in a faster the search mechanism and in an increase in the association rate of TFs to cognate sites. More generally, some studies are starting to clarify the role of nuclear organization in guiding TFs, as detailed below, but how the modulation in chromatin structure observed in physiology (e.g. during development) or in pathology can control the TF search remains largely unexplored.

## What is the role of the nuclear organization in shaping TF dynamics?

The genome is organized at multiple scales, ranging from the 10 nm chromatin fiber scale to the µm scale of chromatin domains, and each of these layers could have a role in the TF search mechanism.

At the nanometric scale, the genome is packaged into nucleosomes, consisting of 147 bp of DNA and core histone proteins whose tails act as substrates for post-translational modifications. A large portion of the genome is wrapped around nucleosomes, which might act as an obstacle for the TF interaction with its target sequences and as roadblocks for 1D diffusion, potentially influencing both the affinity and the search mechanisms of the TFs. The exploration of different TFs, depending on their capability of binding nucleosome-rich regions (e.g. pioneer vs. non-pioneer TFs) could be differently affected by the compaction of chromatin. Three different studies in yeasts analyzed the role of chromatin remodeling complexes (RSC, that displace nucleosomes along the chromatin fiber) on TF targeting. Nucleosomes affect the pioneer TF Rap1 only in terms of its residence time at target sites, with no effect on its search time [62]. Differently, both the inducible TF Ace1 [63]and the PIC subunits [44] respond to the activity of RSC complex by increasing both their search efficiency and the affinity for their targets. Similar observations are made in higher eukaryotes. In Drosophila embryos the pioneer TF Zelda leads to an increased search efficiency of the Bicoid TF [26], while in pluripotent mammalian cells Sox2 and Oct4 can facilitate each other in targeting their binding sites, possibly in a locus specific fashion [64,65].

At the sub-Mb scale the chromatin fiber folds in the so-called topologically associated domains (TADs), that bring similarly regulated distal genomic regions to be in close contact. The identification of TADs mostly derives from biochemical bulk techniques such as Hi-C and their dynamics, heterogeneity across the cell population and function are a matter of current intense investigation. Most notably, TADs containing active genes are thought to generate chromatin loops connecting *cis*-regulatory elements such as promoters to distal regulatory elements such as enhancers located hundreds of kilobases apart. These 3D interactions might generate active chromatin hubs, where high local concentrations of TFs, co-factors and polymerases can activate transcription. The role of TADs on the search mechanisms of TFs has been mostly characterized from a computational point of view, with both coarse-grained [66] and molecular dynamics simulation [67] indicating that genomic regions at low chromatin compaction but high connectivity (e.g. engaged in chromosome loops) are more efficiently targeted by TFs sliding on DNA.

At the micron-scale, the eukaryotic nucleus displays clear spatial compartmentalization between transcriptionally active regions (euchromatin) and denser inactive regions (heterochromatin). From the original description by Emil Heitz in 1928, the euchromatin/heterochromatin model has evolved to include information about the histone modifications characterizing the two chromatin flavors and to relate with the classification into A/B compartments provided by Hi-C data [68]. From a microscopic point of view, the ANC/INC network model, conceived by Thomas and Christoph Cremer, provides a modern representation of the euchromatin/heterochromatin dichotomy [69]. According to this model, the active nuclear compartment (ANC) is pervaded by a three-dimensional network of channels, the interchromatin compartment (IC), which starts from the nuclear pores and branches towards the internal part of the nucleus. One of the IC functions is to provide routes for importing and channeling TFs, co-factors, polymerases, to allow transcription, splicing and DNA repair to occur. Conversely, the inactive nuclear compartment (INC) is formed by highly compacted chromatin organized in chromosome domains (CDs), remotely located from the IC routes, is largely transcriptionally repressed and loosely corresponds to heterochromatin. Repositioning of a gene that needs to be transcribed from the INC to the ANC could facilitate its targeting by TFs. This last proposition of the model is supported by recent high-resolution microscopy data in fixed cells, showing that nascent transcription is enriched in a 100 nm tick-interface between CDs and ICs [70], and that active genomic loci often localize at the boundaries of nuclear sub-compartments [71]. Whether indeed the IC can route nuclear proteins towards its destination is still awaiting for a formal demonstration. Remarkably, however, recent SMT data on gene-editing enzymes TALEN and Cas9 display that the mesoscale organization of chromatin can affect the search mechanism in a protein-specific fashion, and that different search strategies result in different activities of the two enzymes in euchromatin vs heterochromatin [72].

Finally, as intranuclear motion of at least some TFs appears partially confined [43,44,51,73], a point to be addressed is which nuclear compartments can trap a TF within a limited region of the space. Of particular interest are the phase-separated condensates, since they appear as defined compartments with selective permeability. Phase-separated condensates arise from multivalent interactions between proteins or proteins and RNAs [74,75]. Condensates associated with transcription typically contain TFs, coactivators and PolII [76,77] and their formation is often mediated by intrinsically disordered domains (IDRs) [78,79]. It has been proposed that macromolecular condensates and phase-separated compartments in the nucleus could provide a mean to guide nuclear proteins to their targets: by alternating between exhaustive compact exploration within the condensate with fast diffusion outside condensates, the search mechanism would be enhanched, as observed for chromatin regulators [51,80]. For TFs, this increase in the association kinetics, would lead to more robust assembly of the transcription complex and to the up-regulation of transcription. However, recent data by the Rippe's lab is challenging this perspective [56]: by combining live-cell monitoring of transcription and optogenetic control of condensate formation, the authors highlighted that while TFs with IDRs result in higher transcriptional rates, cells with and without condensates show comparable transcriptional activity. Multivalent-interactions mediated between the TF IDR and the nuclear environment [43], rather than the formation of self-condensates, might therefore be responsible for guiding the TF search process and for the activation of the target genes.

## Perspectives

- In the last decade we have accumulated scattered information on how eukaryotic TFs search for their target sites. Whether shared modes of nuclear exploration exist and what are the chromatin/nuclear features controlling such search remain open questions. More studies are also necessary to understand if the parameters governing nuclear exploration are regulated during development and if they are perturbed in disease, although some examples of both instances exist [61,81,82].
- The full characterization of the TF search will arguably benefit from innovative microscopy approaches, including methods to follow single molecules in 3D for a prolonged time. Microscopy techniques based on ‘imaging with zeros' such as MINFLUX or MINSTED [83,84], chemical optimization of fluorescent dyes used for SMT [85,86], or the use of non-fluorescence based single-molecule techniques [87] could all contribute to this scope. Similarly, the impact of different nuclear features/compartments on TF diffusion could be explored by combining SMT with high- spatial and temporal resolution maps of such features, a possibility that has been used only in a handful of studies [51,88].
- Functionally, the impact of the nuclear environment on the TF search could be addressed on systems where chromatin organization is deeply modulated, for example in rod cells of nocturnal animals, featuring heterochromatin inversion [89], during differentiation of pluripotent cells or in those disease models that display genome-wide perturbation of chromatin organization, such as progeria [90]. Combining SMT analysis of TF diffusion with high-throughput analysis of gene positioning in these models could help us understanding how target selectivity is achieved in living cells.

## Abbreviations

ANC: active nuclear compartment
CDs: chromosome domains
GTFs: general transcription factors
IC: interchromatin compartment
IDRs: intrinsically disordered domains
INC: inactive nuclear compartment
MSD: mean squared displacement
PIC: preinitiaton complex
SMT: single molecule tracking
TADs: topologically associated domains
TFs: transcription factors

## References

[1] Woringer, M. and Darzacq, X. (2018) Protein motion in the nucleus: from anomalous diffusion to weak interactions. Biochem. Soc. Trans. 46, 945–956 10.1042/BST20170310 30065106 PMC6103463

[2] Esadze, A. and Stivers, J.T. (2018) Facilitated diffusion mechanisms in DNA base excision repair and transcriptional activation. Chem. Rev. 118, 11298–11323 10.1021/acs.chemrev.8b00513 30379068 PMC6504930

[3] Jana, T., Brodsky, S. and Barkai, N. (2021) Speed–specificity trade-offs in the transcription factors search for their genomic binding sites. Trends Genet. 37, 421–432 10.1016/j.tig.2020.12.001 33414013

[4] Berg, O., Winter, R.B. and von Hippel, P.V. (1981) Diffusion-driven mechanisms of protein translocation on nucleic acids. 1. Models and theory. Biochemistry 20, 6929–6948 10.1021/bi00527a028 7317363

[5] Halford, S.E. and Marko, J.F. (2004) How do site-specific DNA-binding proteins find their targets? Nucleic Acids Res. 32, 3040–3052 10.1093/nar/gkh624 15178741 PMC434431

[6] Mirny, L., Slutsky, M., Wunderlich, Z., Tafvizi, A., Leith, J. and Kosmrlj, A. (2009) How a protein searches for its site on DNA: the mechanism of facilitated diffusion. J. Phys. Math. Theor. 42, 434013 10.1088/1751-8113/42/43/434013

[7] Suter, D.M. (2020) Transcription factors and DNA play hide and seek. Trends Cell Biol. 30, 491–500 10.1016/j.tcb.2020.03.003 32413318

[8] De March, M., Merino, N., Barrera-Vilarmau, S., Crehuet, R., Onesti, S., Blanco, F.J. et al. (2017) Structural basis of human PCNA sliding on DNA. Nat. Commun. 8, 13935 10.1038/ncomms13935 28071730 PMC5234079

[9] Silverstein, T.D., Gibb, B. and Greene, E.C. (2014) Visualizing protein movement on DNA at the single-molecule level using DNA curtains. DNA Repair 20, 94–109 10.1016/j.dnarep.2014.02.004 24598576 PMC4111998

[10] Cravens, S.L., Schonhoft, J.D., Rowland, M.M., Rodriguez, A.A., Anderson, B.G. and Stivers, J.T. (2015) Molecular crowding enhances facilitated diffusion of two human DNA glycosylases. Nucleic Acids Res. 43, 4087–4097 10.1093/nar/gkv301 25845592 PMC4417188

[11] Liu, Y., Lagowski, J.P., Vanderbeek, G.E. and Kulesz-Martin, M.F. (2004) Facilitated search for specific genomic targets by p53 C-terminal basic DNA binding domain. Cancer Biol. Ther. 3, 1102–1108 10.4161/cbt.3.11.1189 15467437

[12] Elf, J., Li, G.-W. and Xie, X.S. (2007) Probing transcription factor dynamics at the single-molecule level in a living cell. Science 316, 1191–1194 10.1126/science.1141967 17525339 PMC2853898

[13] Hammar, P., Leroy, P., Mahmutovic, A., Marklund, E.G. Berg, O.G. and Elf, J. (2012) The lac repressor displays facilitated diffusion in living cells. Science 336, 1595–1598 10.1126/science.1221648 22723426

[14] Stracy, M., Schweizer, J., Sherratt, D.J., Kapanidis, A.N., Uphoff, S. and Lesterlin, C. (2021) Transient non-specific DNA binding dominates the target search of bacterial DNA-binding proteins. Mol. Cell 81, 1499–1514.e6 10.1016/j.molcel.2021.01.039 33621478 PMC8022225

[15] Zobeck, K.L., Buckley, M.S., Zipfel, W.R. and Lis, J.T. (2010) Recruitment timing and dynamics of transcription factors at the Hsp70 loci in living cells. Mol. Cell 40, 965–975 10.1016/j.molcel.2010.11.022 21172661 PMC3021954

[16] Castellanos, M., Mothi, N. and Muñoz, V. (2020) Eukaryotic transcription factors can track and control their target genes using DNA antennas. Nat. Commun. 11, 540 10.1038/s41467-019-14217-8 31992709 PMC6987225

[17] Yu, J. (2016) Single-molecule studies in live cells. Annu. Rev. Phys. Chem. 67, 565–585 10.1146/annurev-physchem-040215-112451 27070321

[18] Elf, J. and Barkefors, I. (2019) Single-molecule kinetics in living cells. Annu. Rev. Biochem. 88, 635–659 10.1146/annurev-biochem-013118-110801 30359080

[19] Jaqaman, K., Loerke, D., Mettlen, M., Kuwata, H., Grinstein, S., Schmid, S.L. et al. (2008) Robust single-particle tracking in live-cell time-lapse sequences. Nat. Methods 5, 695–702 10.1038/nmeth.1237 18641657 PMC2747604

[20] Chenouard, N., Smal, I., de Chaumont, F., Maška, M., Sbalzarini, I.F., Gong, Y. et al. (2014) Objective comparison of particle tracking methods. Nat. Methods 11, 281–289 10.1038/nmeth.2808 24441936 PMC4131736

[21] Gebhardt, J.C.M., Suter, D.M., Roy, R., Zhao, Z.W., Chapman, A.R., Basu, S. et al. (2013) Single-molecule imaging of transcription factor binding to DNA in live mammalian cells. Nat. Methods 10, 421–426 10.1038/nmeth.2411 23524394 PMC3664538

[22] Mazza, D., Abernathy, A., Golob, N., Morisaki, T. and McNally, J.G. (2012) A benchmark for chromatin binding measurements in live cells. Nucleic Acids Res. 40, e119 10.1093/nar/gks701 22844090 PMC3424588

[23] Speil, J., Baumgart, E., Siebrasse, J.-P., Veith, R., Vinkemeier, U. and Kubitscheck, U. (2011) Activated STAT1 transcription factors conduct distinct saltatory movements in the cell nucleus. Biophys. J. 101, 2592–2600 10.1016/j.bpj.2011.10.006 22261046 PMC3297778

[24] Grimm, J.B., English, B.P., Chen, J., Slaughter, J.P., Zhang, Z., Revyakin, A. et al. (2015) A general method to improve fluorophores for live-cell and single-molecule microscopy. Nat. Methods 12, 244–250 10.1038/nmeth.3256 25599551 PMC4344395

[25] Grimm, J.B., English, B.P., Choi, H., Muthusamy, A.K., Mehl, B.P., Dong, P. et al. (2016) Bright photoactivatable fluorophores for single-molecule imaging. Nat. Methods 13, 985–988 10.1038/nmeth.4034 27776112

[26] Mir, M., Reimer, A., Haines, J.E., Li, X.-Y., Stadler, M., Garcia, H. et al. (2017) Dense Bicoid hubs accentuate binding along the morphogen gradient. Genes Dev. 31, 1784–1794 10.1101/gad.305078.117 28982761 PMC5666676

[27] Tokunaga, M., Imamoto, N. and Sakata-Sogawa, K. (2008) Highly inclined thin illumination enables clear single-molecule imaging in cells. Nat. Methods 5, 159–161 10.1038/nmeth1171 18176568

[28] Hansen, A.S., Woringer, M., Grimm, J.B., Lavis, L.D., Tjian, R. and Darzacq, X. (2018) Robust model-based analysis of single-particle tracking experiments with spot-On. eLife 7, e33125 10.7554/eLife.33125 29300163 PMC5809147

[29] Chen, J., Zhang, Z., Li, L., Chen, B.-C., Revyakin, A., Hajj, B. et al. (2014) Single-Molecule dynamics of enhanceosome assembly in embryonic stem cells. Cell 156, 1274–1285 10.1016/j.cell.2014.01.062 24630727 PMC4040518

[30] Paakinaho, V., Presman, D.M., Ball, D.A., Johnson, T.A., Schiltz, R.L., Levitt, P. et al. (2017) Single-molecule analysis of steroid receptor and cofactor action in living cells. Nat. Commun. 8, 15896 10.1038/ncomms15896 28635963 PMC5482060

[31] Nagashima, R., Hibino, K., Ashwin, S.S., Babokhov, M., Fujishiro, S., Imai, R. et al. (2019) Single nucleosome imaging reveals loose genome chromatin networks via active RNA polymerase II. J. Cell Biol. 218, 1511–1530 10.1083/jcb.201811090 30824489 PMC6504897

[32] Persson, F., Lindén, M., Unoson, C. and Elf, J. (2013) Extracting intracellular diffusive states and transition rates from single-molecule tracking data. Nat. Methods 10, 265–269 10.1038/nmeth.2367 23396281

[33] Vega, A.R., Freeman, S.A., Grinstein, S. and Jaqaman, K. (2018) Multistep track segmentation and motion classification for transient mobility analysis. Biophys. J. 114, 1018–1025 10.1016/j.bpj.2018.01.012 29539390 PMC5883548

[34] Granik, N., Weiss, L.E., Nehme, E., Levin, M., Chein, M., Perlson, E. et al. (2019) Single-Particle diffusion characterization by deep learning. Biophys. J. 117, 185–192 10.1016/j.bpj.2019.06.015 31280841 PMC6701009

[35] Callegari, A., Sieben, C., Benke, A., Suter, D.M., Fierz, B., Mazza, D. et al. (2019) Single-molecule dynamics and genome-wide transcriptomics reveal that NF-kB (p65)-DNA binding times can be decoupled from transcriptional activation. PLoS Genet. 15, e1007891 10.1371/journal.pgen.1007891 30653501 PMC6353211

[36] Hansen, A.S., Pustova, I., Cattoglio, C., Tjian, R. and Darzacq, X. (2017) CTCF and cohesin regulate chromatin loop stability with distinct dynamics. eLife 6, e25776 10.7554/eLife.25776 28467304 PMC5446243

[37] Garcia, D.A., Fettweis, G., Presman, D.M., Paakinaho, V., Jarzynski, C., Upadhyaya, A. et al. (2021) Power-law behaviour of transcription factor dynamics at the single-molecule level implies a continuum affinity model. Nucleic Acids Res. 10.1093/nar/gkab072 PMC8266587 33592625

[38] Clauß, K., Popp, A.P., Schulze, L., Hettich, J., Reisser, M., Escoter Torres, L. et al. (2017) DNA residence time is a regulatory factor of transcription repression. Nucleic Acids Res. 45, 11121–11130 10.1093/nar/gkx728 28977492 PMC5737411

[39] Loffreda, A., Jacchetti, E., Antunes, S., Rainone, P., Daniele, T., Morisaki, T. et al. (2017) Live-cell p53 single-molecule binding is modulated by C-terminal acetylation and correlates with transcriptional activity. Nat. Commun. 8, 313 10.1038/s41467-017-00398-7 28827596 PMC5567047

[40] Swinstead, E.E., Miranda, T.B., Paakinaho, V., Baek, S., Goldstein, I., Hawkins, M. et al. (2016) Steroid receptors reprogram FoxA1 occupancy through dynamic chromatin transitions. Cell 165, 593–605 10.1016/j.cell.2016.02.067 27062924 PMC4842147

[41] Reisser, M., Hettich, J., Kuhn, T., Popp, A.P., Große-Berkenbusch, A. and Gebhardt, J.C.M. (2020) Inferring quantity and qualities of superimposed reaction rates from single molecule survival time distributions. Sci. Rep. 10, 1758 10.1038/s41598-020-58634-y 32019978 PMC7000831

[42] Caccianini, L., Normanno, D., Izeddin, I. and Dahan, M. (2015) Single molecule study of non-specific binding kinetics of LacI in mammalian cells. Faraday Discuss 184, 393–400 10.1039/C5FD00112A 26387491

[43] Garcia, D.A., Johnson, T.A., Presman, D.M., Fettweis, G., Wagh, K., Rinaldi, L. et al. (2021) An intrinsically disordered region-mediated confinement state contributes to the dynamics and function of transcription factors. Mol. Cell 81, 1484–1498.e6 10.1016/j.molcel.2021.01.013 33561389 PMC9258326

[44] Nguyen, V.Q., Ranjan, A., Liu, S., Tang, X., Ling, Y.H., Wisniewski, J. et al. (2020) Spatio-temporal coordination of transcription preinitiation complex assembly in live cells. bioRxiv 10.1101/2020.12.30.424853 PMC8420877 34375585

[45] Tatavosian, R., Kent, S., Brown, K., Yao, T., Duc, H.N., Huynh, T.N. et al. (2019) Nuclear condensates of the polycomb protein chromobox 2 (CBX2) assemble through phase separation. J. Biol. Chem. 294, 1451–1463 10.1074/jbc.RA118.006620 30514760 PMC6364756

[46] Saxton, M.J. (1994) Anomalous diffusion due to obstacles: a monte carlo study. Biophys. J. 66, 394–401 10.1016/S0006-3495(94)80789-1 8161693 PMC1275707

[47] Mueller, F., Stasevich, T.J., Mazza, D. and McNally, J.G. (2013) Quantifying transcription factor kinetics: At work or at play? Crit. Rev. Biochem. Mol. Biol. 48, 492–514 10.3109/10409238.2013.833891 24025032

[48] Raccaud, M., Friman, E.T., Alber, A.B., Agarwal, H., Deluz, C., Kuhn, T. et al. (2019) Mitotic chromosome binding predicts transcription factor properties in interphase. Nat. Commun. 10, 487 10.1038/s41467-019-08417-5 30700703 PMC6353955

[49] Bénichou, O., Chevalier, C., Klafter, J., Meyer, B. and Voituriez, R. (2010) Geometry-controlled kinetics. Nat. Chem. 2, 472–477 10.1038/nchem.622 20489716

[50] Meyer, B., Bénichou, O., Kafri, Y. and Voituriez, R. (2012) Geometry-induced bursting dynamics in gene expression. Biophys. J. 102, 2186–2191 10.1016/j.bpj.2012.03.060 22824283 PMC3341560

[51] Hansen, A.S., Amitai, A., Cattoglio, C., Tjian, R. and Darzacq, X. (2020) Guided nuclear exploration increases CTCF target search efficiency. Nat. Chem. Biol. 16, 257–266 10.1038/s41589-019-0422-3 31792445 PMC7036004

[52] Récamier, V., Izeddin, I., Bosanac, L., Dahan, M., Proux, F. and Darzacq, X. (2014) Single cell correlation fractal dimension of chromatin. Nucleus 5, 75–84 10.4161/nucl.28227 24637833 PMC4028358

[53] Bancaud, A., Huet, S., Daigle, N., Mozziconacci, J., Beaudouin, J. and Ellenberg, J. (2009) Molecular crowding affects diffusion and binding of nuclear proteins in heterochromatin and reveals the fractal organization of chromatin. EMBO J. 28, 3785–3798 10.1038/emboj.2009.340 19927119 PMC2797059

[54] Izeddin, I., Récamier, V., Bosanac, L., Cissé, I.I., Boudarene, L., Dugast-Darzacq, C. et al. (2014) Single-molecule tracking in live cells reveals distinct target-search strategies of transcription factors in the nucleus. eLife 3, e02230 10.7554/eLife.02230 PMC4095940 24925319

[55] Tafvizi, A., Huang, F., Fersht, A.R., Mirny, L.A. and van Oijen, A.M. (2011) A single-molecule characterization of p53 search on DNA. Proc. Natl Acad. Sci. U.S.A. 108, 563–568 10.1073/pnas.1016020107 21178072 PMC3021058

[56] Trojanowski, J., Frank, L., Rademacher, A., Grigaitis, P. and Rippe, K. (2021) Transcription activation is enhanced by multivalent interactions independent of liquid-liquid phase separation. bioRxiv 10.1101/2021.01.27.428421 35537448

[57] Vuzman, D. and Levy, Y. (2010) DNA search efficiency is modulated by charge composition and distribution in the intrinsically disordered tail. Proc. Natl Acad. Sci. U.S.A. 107, 21004–21009 10.1073/pnas.1011775107 21078959 PMC3000266

[58] Filtz, T.M., Vogel, W.K. and Leid, M. (2014) Regulation of transcription factor activity by interconnected post-translational modifications. Trends Pharmacol. Sci. 35, 76–85 10.1016/j.tips.2013.11.005 24388790 PMC3954851

[59] Vuzman, D. and Levy, Y. (2012) Intrinsically disordered regions as affinity tuners in protein-DNA interactions. Mol. Biosyst. 8, 47–57 10.1039/C1MB05273J 21918774

[60] Louphrasitthiphol, P., Siddaway, R., Loffreda, A., Pogenberg, V., Friedrichsen, H., Schepsky, A. et al. (2020) Tuning transcription factor availability through acetylation-Mediated genomic redistribution. Mol. Cell 79, 472–487.e10 10.1016/j.molcel.2020.05.025 32531202 PMC7427332

[61] Reisser, M., Palmer, A., Popp, A.P., Jahn, C., Weidinger, G. and Gebhardt, J.C.M. (2018) Single-molecule imaging correlates decreasing nuclear volume with increasing TF-chromatin associations during zebrafish development. Nat. Commun. 9, 5218 10.1038/s41467-018-07731-8 30523256 PMC6283880

[62] Mivelaz, M., Cao, A.-M., Kubik, S., Zencir, S., Hovius, R., Boichenko, I. et al. (2020) Chromatin fiber invasion and nucleosome displacement by the Rap1 transcription factor. Mol. Cell 77, 488–500.e9 10.1016/j.molcel.2019.10.025 31761495 PMC7005674

[63] Mehta, G.D., Ball, D.A., Eriksson, P.R., Chereji, R.V., Clark, D.J., McNally, J.G. et al. (2018) Single-Molecule analysis reveals linked cycles of RSC chromatin remodeling and Ace1p transcription factor binding in yeast. Mol. Cell 72, 875–887.e9 10.1016/j.molcel.2018.09.009 30318444 PMC6289719

[64] Biddle, J.W., Nguyen, M. and Gunawardena, J. (2019) Negative reciprocity, not ordered assembly, underlies the interaction of Sox2 and Oct4 on DNA. eLife 8, e41017 10.7554/eLife.41017 30762521 PMC6375704

[65] Friman, E.T., Deluz, C., Meireles-Filho, A.C., Govindan, S., Gardeux, V., Deplancke, B. et al. (2019) Dynamic regulation of chromatin accessibility by pluripotency transcription factors across the cell cycle. eLife 8, e50087 10.7554/eLife.50087 31794382 PMC6890464

[66] Avcu, N. and Molina, N. (2016) Chromatin structure shapes the search process of transcription factors. bioRxiv 10.1101/050146

[67] Cortini, R. and Filion, G.J. (2018) Theoretical principles of transcription factor traffic on folded chromatin. Nat. Commun. 9, 1740 10.1038/s41467-018-04130-x 29712907 PMC5928121

[68] Jost, K.L., Bertulat, B. and Cardoso, M.C. (2012) Heterochromatin and gene positioning: inside, outside, any side? Chromosoma 121, 555–563 10.1007/s00412-012-0389-2 23090282 PMC3501169

[69] Cremer, T., Cremer, M., Hübner, B., Silahtaroglu, A., Hendzel, M., Lanctôt, C. et al. (2020) The interchromatin compartment participates in the structural and functional organization of the cell nucleus. Bioessays 42, 1900132 10.1002/bies.201900132 31994771

[70] Miron, E., Oldenkamp, R., Brown, J.M., Pinto, D.M.S., Xu, C.S., Faria, A.R. et al. (2020) Chromatin arranges in chains of mesoscale domains with nanoscale functional topography independent of cohesin. Sci. Adv. 6, eaba8811 10.1126/sciadv.aba8811 32967822 PMC7531892

[71] Takei, Y., Yun, J., Zheng, S., Ollikainen, N., Pierson, N., White, J. et al. (2021) Integrated spatial genomics reveals global architecture of single nuclei. Nature 590, 344–350 10.1038/s41586-020-03126-2 33505024 PMC7878433

[72] Jain, S., Shukla, S., Yang, C., Zhang, M., Fatma, Z., Lingamaneni, M. et al. (2021) TALEN outperforms Cas9 in editing heterochromatin target sites. Nat. Commun. 12, 606 10.1038/s41467-020-20672-5 33504770 PMC7840734

[73] Lerner, J., Gomez-Garcia, P.A., McCarthy, R.L., Liu, Z., Lakadamyali, M. and Zaret, K.S. (2020) Two-parameter mobility assessments discriminate diverse regulatory factor behaviors in chromatin. Mol. Cell 79, 677–688.e6 10.1016/j.molcel.2020.05.036 32574554 PMC7483934

[74] Alberti, S., Gladfelter, A. and Mittag, T. (2019) Considerations and challenges in studying liquid-liquid phase separation and biomolecular condensates. Cell 176, 419–434 10.1016/j.cell.2018.12.035 30682370 PMC6445271

[75] Riback, J.A., Zhu, L., Ferrolino, M.C., Tolbert, M., Mitrea, D.M., Sanders, D.W. et al. (2020) Composition-dependent thermodynamics of intracellular phase separation. Nature 581, 209–214 10.1038/s41586-020-2256-2 32405004 PMC7733533

[76] Sabari, B.R., Dall'Agnese, A., Boija, A., Klein, I.A., Coffey, E.L., Shrinivas, K. et al. (2018) Coactivator condensation at super-enhancers links phase separation and gene control. Science 361, eaar3958 10.1126/science.aar3958 29930091 PMC6092193

[77] Lu, Y., Wu, T., Gutman, O., Lu, H., Zhou, Q., Henis, Y.I. et al. (2020) Phase separation of TAZ compartmentalizes the transcription machinery to promote gene expression. Nat. Cell Biol. 22, 453–464 10.1038/s41556-020-0485-0 32203417 PMC11044910

[78] Chong, S., Dugast-Darzacq, C., Liu, Z., Dong, P., Dailey, G.M., Cattoglio, C. et al. (2018) Imaging dynamic and selective low-complexity domain interactions that control gene transcription. Science 361, eaar2555 10.1126/science.aar2555 29930090 PMC6961784

[79] Boija, A., Klein, I.A., Sabari, B.R., Dall'Agnese, A., Coffey, E.L., Zamudio, A.V. et al. (2018) Transcription factors activate genes through the phase-separation capacity of their activation domains. Cell 175, 1842–1855.e16 10.1016/j.cell.2018.10.042 30449618 PMC6295254

[80] Kent, S., Brown, K., Yang, C., Alsaihati, N., Tian, C., Wang, H. et al. (2020) Phase-Separated transcriptional condensates accelerate target-search process revealed by live-cell single-molecule imaging. Cell Rep. 33, 108248 10.1016/j.celrep.2020.108248 33053359 PMC7593837

[81] Li, L., Liu, H., Dong, P., Li, D., Legant, W.R., Grimm, J.B. et al. (2016) Real-time imaging of Huntingtin aggregates diverting target search and gene transcription. eLife 5, e17056 10.7554/eLife.17056 27484239 PMC4972539

[82] White, M.D., Angiolini, J.F., Alvarez, Y.D., Kaur, G., Zhao, Z.W., Mocskos, E. et al. (2016) Long-lived binding of Sox2 to DNA predicts cell fate in the four-Cell mouse embryo. Cell 165, 75–87 10.1016/j.cell.2016.02.032 27015308

[83] Gwosch, K.C., Pape, J.K., Balzarotti, F., Hoess, P., Ellenberg, J., Ries, J. et al. (2020) MINFLUX nanoscopy delivers 3D multicolor nanometer resolution in cells. Nat. Methods 17, 217–224 10.1038/s41592-019-0688-0 31932776

[84] Weber, M., Leutenegger, M., Stoldt, S., Jakobs, S., Mihaila, T.S., Butkevich, A.N. et al. (2020) MINSTED fluorescence localization and nanoscopy. BioRxiv 10.1101/2020.10.31.363424 PMC7610723 33953795

[85] Tsunoyama, T.A., Watanabe, Y., Goto, J., Naito, K., Kasai, R.S., Suzuki, K.G.N. et al. (2018) Super-long single-molecule tracking reveals dynamic-anchorage-induced integrin function. Nat. Chem. Biol. 14, 497–506 10.1038/s41589-018-0032-5 29610485

[86] Liu, H., Dong, P., Ioannou, M.S., Li, L., Shea, J., Pasolli, H.A. et al. (2018) Visualizing long-term single-molecule dynamics in vivo by stochastic protein labeling. Proc. Natl Acad. Sci. U.S.A. 115, 343–348 10.1073/pnas.1713895115 29284749 PMC5777047

[87] Taylor, R.W., Mahmoodabadi, R.G., Rauschenberger, V., Giessl, A., Schambony, A. and Sandoghdar, V. (2019) Interferometric scattering microscopy reveals microsecond nanoscopic protein motion on a live cell membrane. Nat. Photonics 13, 480–487 10.1038/s41566-019-0414-6

[88] Liu, Z., Legant, W.R., Chen, B.-C., Li, L., Grimm, J.B., Lavis, L.D. et al. (2014) 3D imaging of Sox2 enhancer clusters in embryonic stem cells. eLife 3, e04236 10.7554/eLife.04236 25537195 PMC4381973

[89] Falk, M., Feodorova, Y., Naumova, N., Imakaev, M., Lajoie, B.R., Leonhardt, H. et al. (2019) Heterochromatin drives compartmentalization of inverted and conventional nuclei. Nature 570, 395–399 10.1038/s41586-019-1275-3 31168090 PMC7206897

[90] Sebestyén, E., Marullo, F., Lucini, F., Petrini, C., Bianchi, A., Valsoni, S., et al. (2020) SAMMY-seq reveals early alteration of heterochromatin and deregulation of bivalent genes in Hutchinson-Gilford progeria syndrome. Nat. Commun. 11, 6274 10.1038/s41467-020-20048-9 33293552 PMC7722762

